# Hybrid Molecularly Imprinted Polymers: The Future of Nanomedicine?

**DOI:** 10.3390/nano11113091

**Published:** 2021-11-16

**Authors:** Maylis Garnier, Michèle Sabbah, Christine Ménager, Nébéwia Griffete

**Affiliations:** 1 PHysico-Chimie des Electrolytes et Nanosystèmes InterfaciauX (PHENIX), Sorbonne Université, CNRS, 4 Place Jussieu, F-75005 Paris, France; maylis.garnier@sorbonne-universite.fr; 2 Saint-Antoine Research Center (CRSA), INSERM, CNRS, Sorbonne Université, F-75012 Paris, France; michele.sabbah@inserm.fr

**Keywords:** nanomedicine, molecularly imprinted polymer, drug delivery, targeting, hybrid material

## Abstract

Molecularly imprinted polymers (MIPs) have been widely used in nanomedicine in the last few years. However, their potential is limited by their intrinsic properties resulting, for instance, in lack of control in drug release processes or complex detection for in vivo imaging. Recent attempts in creating hybrid nanomaterials combining MIPs with inorganic nanomaterials succeeded in providing a wide range of new interesting properties suitable for nanomedicine. Through this review, we aim to illustrate how hybrid molecularly imprinted polymers may improve patient care with enhanced imaging, treatments, and a combination of both.

## 1. Introduction

The application of nanotechnologies to medicine, or for short, nanomedicine, is predicted to revolutionize the future of healthcare. When the size of materials is decreased to nanoscale, an abundance of unique characteristics is newly displayed: large surface-to-mass ratio and small volumes facilitating cellular uptake and intracellular transport. Nanoparticles (NPs) tend to accumulate in tumors due to a physiologically-based phenomenon: the enhanced permeability—of the endothelial lining of the blood vessel—and retention (EPR) effect. This passive targeting has been widely employed to treat tumors using drug delivery systems [1,2,3,4] or heat-generating nanosystems [5]. Not limited to cancer therapy, with interesting developments to treat burns [6] or achieve antimicrobial effects [7], those systems are composed of a wide range of materials: gold, silver, polymers, silica, iron oxide, etc., each employed for its interesting specific property.

The most recent developments in nanomedicine aim at a personalization of the treatments and hence require an active targeting of biological markers. Such targeting is performed through the functionalization with antibodies [8,9], fragments of antibodies [10], or aptamers [11,12,13,14] in order to increase the efficiency of immunotherapies and prediction of therapeutic response [15,16]. It is only recently that molecularly imprinted polymers (MIPs) have been used to enhance the targeting properties of nanoparticles. Another aspect developed in nanomedicine is the possibility to use nanosized objects to transport and deliver drugs at a given location inside a living body, despite biological barriers and degradation mechanisms. For this purpose, it is also possible to use nanosized polymer materials and to render them more efficient through molecular imprinting.

**Figure 1:**
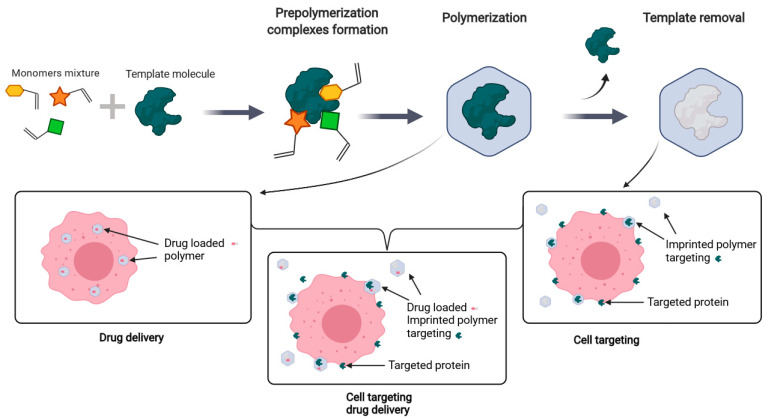
MIPs synthesis principle. The target used as a template is added to a polymer mixture, forming pre-polymerization complexes. After the initiation of the polymerization, the template is embedded inside the polymer matrix and can be removed, forming highly specific cavities. In case of drug delivery applications, the polymer can be reloaded. The imprinting process enables an efficient targeting through a surface protein that can be combined with drug loading of the material. Created with BioRender.com the 3 April 2021.

Molecular imprinting is a method inducing molecular recognition properties during the formation of the three-dimensional structure of a polymer in the presence of a template molecule. After the extraction of the molecule, the polymer matrix contains tailor-made binding sites, cavities complementary in size, shape, and functionality to the template that can recognize the targeted compound with high selectivity (Figure 1).

**Figure 2:**
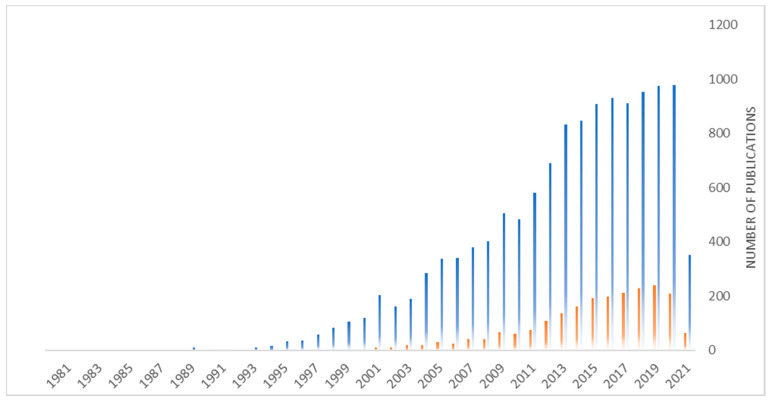
Evolution of the number of publications related to molecularly imprinted polymers (blue) and the combination of molecularly imprinted polymers and any of the following keyword: hybrids, nanocomposites, gold, silver, iron, silica, quantum dots, carbon dots (orange). Data extracted from 1findr.1science.com the 20 April 2021.

In contrast to biological antibodies, the synthesis of molecularly imprinted polymers is reproducible, fast, not involving animals, and relatively economical. They are very stable physically and chemically [17,18,19], not degraded by proteases or denatured by solvents, and can be engineered and tailored for any given application. Moreover, they are notable for their good biocompatibility [20], solubility, ability to cross the cell membrane [20], and lack of immunogenic response [20]. Nanosized MIPs (nanoMIPs) have larger surface/mass ratio, more easily accessible recognition sites, lower heterogeneities, and better solubilities than micrometric MIPs. Due to these remarkable properties, there has been a growing interest concerning the use of nanoMIPs for medical purposes for the past decades. Now, a growing trend in MIPs-related research is their combination with inorganic materials, enlarging the range of possible applications (Figure 2). Inorganic nanoparticles, by possessing large surface-to-volume ratio and size-related physical and chemical properties, are good candidates to support molecular imprinting polymer at their surface. These MIP nanomaterials enable complete removal of templates after the synthesis, enhanced site accessibility, and have a well-defined shape. The first superparamagnetic MIP composite beads incorporating Fe_3_O_4_ were prepared more than two decades ago using suspension polymerization [21]. It is only recently that the use of superparamagnetic MIP became important in nanomedicine as a result of their interesting physical intrinsic properties [22,23]. The imprinting of molecules can also be achieved using as a silica nanoparticles as a support [24]. Carbon nanotubes have extraordinary mechanical, electrical, and thermal properties, on top of being biocompatible [25]. These materials are also used as supports for surface imprinting of proteins and their unique properties can be exploited for medical applications. Quantum dots, characterized by excellent stability and specific emission wavelengths depending on their size, were pioneered by Brus’ group [26]. This exceptional nanomaterial can be couped to MIPs for useful applications in targeted imaging. In 2020, 980 publications were released containing mentions of “molecularly imprinted polymers” and 209 referred to a combination of MIPs with an inorganic material (Data extracted from 1findr.1science.com the 20 April 2021).

Hence, combining molecularly imprinted polymer with inorganic nanoparticles could lead to a novel class of hybrid materials useful in nanomedicine, given their ability to respond to external physical stimuli.

Recently, some reviews focusing on molecularly imprinted polymer for biological application [27], for cancer therapy [28], or for bioimaging and therapy [29] have been published. One review concerns magnetic molecularly imprinted polymers for targeted cancer therapy [23]. However, none of them focuses on the development of hybrid magnetic MIPs in nanomedicine and their possible contribution to this field.

In this review, we focus on the recent development of nanosized molecularly imprinted particles (nanoMIPs) for medical applications and their improvement through their combination with inorganic nanoparticles (nanoMIPs hybrids).

## 2. Fundamentals about MIPs and Their Adaptation for Nanomedicine

### 2.1. Historical Applications

Molecularly imprinted polymers were first widely employed in the separation field, for analytical purposes. They are often used as a chromatographic stationary phase or to selectively extract trace molecules from complex matrices using the solid phase extraction technique. During this process, the analytes are separated and pre-concentrated, resulting in a lower limit of detection (LOD) than with classical techniques, even in complex samples [30] such as fish, meat [31,32], or milk [33]. This type of system can also be used for the purification and enantioselective separation of drugs [34,35], for example by forming enantioselective membranes [36]. The comparison of MIPs performances for separation purposes with those of immunosorbents and aptamers leads to similar results for each technique when analyzing complex samples [37], which is quite encouraging for future developments of MIPs nanoparticles for nanomedicine.

The good stability of MIPs combined with their low cost and high selectivity toward molecules then led to the development of sensors able to detect chemical compounds. The most challenging aspect of those devices is to transform the MIPs recognition into signals. Label-free sensors have been developed, mostly inspired from DNA chips and immunosensors. Many techniques are available for measuring the signal such as quartz crystal microbalance (QCM), surface acoustic wave (SAW), surface plasmon resonance (SPR) measurements, Raman and Fourier transform infrared spectroscopy (FTIR), and atomic force microscopy (AFM). Those techniques were developed using MIPs as recognition elements and even achieved picomolar limits of detection [38].

Lately, MIPs mimicking biological functions [39] with, for instance, enzyme-like activity [40], have been designed. In 2013, an assay similar to the enzyme-linked immunosorbent assay (ELISA) was developed, replacing the antibodies coating microplate wells with molecularly imprinted polymer. The sensitivity of the assay was three orders of magnitude better than a previously described ELISA assay based on antibodies (2.5 pM), with more stability and easier implementation as no cold-chain logistic was required. In terms of affinity and specificity, MIPs possess binding characteristics similar to those of antibodies or biological receptors, which makes them often described as “synthetic antibodies” [41]. Replacing antibodies with MIPs nanoparticles in ELISA-like and other similar assays has been widely studied, leading to tests with a picomolar LOD similar to antibodies-based assays (LOD for biotin: 1.2 pM for nanoMIPs versus 2.5 pM for the antibody assay, LOD for fumonisin B2: 6.1 pM versus 25 pM) [42,43,44]. Dissociation constants (Kd), depending on the monomers used and the nature of the template, are often in the nanomolar range: 0.48 nM for NanoMIPs against vancomycin [45]. Hence, they might be used for a great number of medical applications that previously required tailor-made antibodies or for which no antibodies could be developed. For example, MIP sensors can directly be applied to diagnose diseases: in 2016, Lieberzeit et al. [46] developed a MIP-based QCM sensor able to specifically trap lipoproteins, a cardiovascular biomarker, as its concentration in human serum is inversely correlated to high risk of coronary disease. This device, applied to human samples, led to similar results to a standard homogenous enzymatic assay.

As MIPs show efficiency in biological sensors, scientists are now interested in their use in the medical field. However, their synthetic process has to be modified and adapted to be compatible with the desired application, with an emphasis on biocompatibility when aiming for in vivo applications.

### 2.2. Synthesis of MIPs

The origin of molecularly imprinted polymers can be traced as far as the 1930s when a Soviet researcher named Polyakov discovered an improvement of the adsorption of benzene derivatives on silica particles when prepared in their presence [47]. During the following years, the study of the formation process of those imprinted polymers led to enhanced recognition properties and in the 1970s, Günter Wulff synthesized an organic polymer able to separate racemates [48]. When designing an imprinted polymer, different imprinting strategies are employed, depending on the application [49], as the morphology of the imprinted polymer can affect recognition properties or potential application [50].

According to the nature of the interaction between the template and functional monomers, three main approaches can be distinguished to classify synthesis techniques of molecularly imprinted polymers: covalent, non-covalent, and semi-covalent techniques. In covalent imprinting, the templates are chemically bound to monomers. The chosen reaction being reversible, for instance, a boronic acid function reacting with diol as employed by Wulff [51], the template is then chemically removed from the polymer, forming highly specific cavities able to recapture the template, either covalently or non-covalently [52] (the latest resulting in what is called the semi-covalent approach). However, this technique is limited by the ability to perform reversible covalent bonds between the template and monomers and to extract the template without disrupting the polymer.

The idea of creating imprinted polymers through non-covalent interactions has been widely explored by Mosbach. The interactions may be ionic, hydrogen bonding, hydrophobic, electrostatic, etc. After a successful attempt with dyes [53] in what was called at the time a “host-guest” polymerization, Mosbach and his team developed an imprinted polymer able to separate amino acid derivatives [54]. With this technique, improved selectivity may be obtained with mixtures of monomers [51]. This method, however, tends to produce polymers with a less homogenous repartition of binding sites than through a covalent approach.

At their origin, MIPs were synthesized through bulk polymerization, forming macroporous polymer networks with the ability to entrap molecules. Those polymers had to be ground to obtain small particles and create a large surface area for recapture. However, this step is time consuming and results in irregular shapes and sizes as well as heterogeneities in the binding sites repartition.

**Figure 3:**
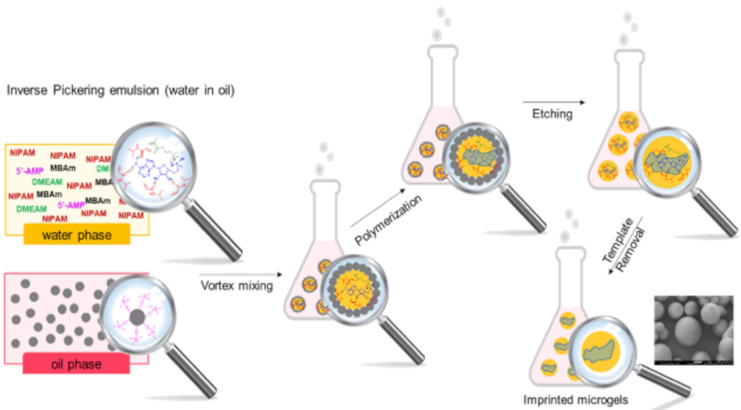
Inverse Pickering emulsion used for the synthesis of MIP-DMEAM-NIPAM-based hydrogel microbeads. Reproduced with permission. [56] © 2021 Elsevier B.V.

Obtaining monodispersed polymer particles is a key condition to improve reproducibility. Conducting the polymerization in a biphasic system is a great option to obtain regular shapes and sizes. The emulsion polymerization technique is usually performed in oil-in-water emulsions with a surfactant. In 2002, Tovar et al. [55] synthesized MIPs nanoparticles by miniemulsion polymerization, allowing direct non-covalent imprinting of a chiral amino acid derivative. The resulting nanospheres, with a size of about 200 nm, showed enantiospecificity. However, the use of surfactants can interfere with molecular recognition, contaminate the polymeric product, and require complicated and time-consuming purification steps. Pickering emulsion polymerization, which consists in the addition of nanoparticles such as silica during the process, enable a surfactant free-polymerization (Figure 3). The process has been employed in the synthesis of imprinted hydrogel microbeads able to passively release the nucleotide adenosine 5’-monophosphate for an application in cosmetology [56].

In 1999, Mosbach et al. [57] first described the precipitation polymerization technique which produces uniform sub-micrometer imprinted particles. The resulting molecularly imprinted microspheres were highly specific for their target, theophylline or 17β-estradiol, and had higher adsorption capacities compared to the particles obtained by grinding an imprinted monolith. This technique has also been successfully employed for many medical purposes such as the design of a transdermal formulation of a MIP with the ability to release encapsulated nicotine [58]. The polymerization process occurs in a solvent excess and stops when the particles reach a critical size which makes them precipitate. However, the dilution tends to decrease interactions between the template and the functional monomers leading to less selectivity.

### 2.3. Synthesis of MIP Nanoparticles for Nanomedicine

While their advantages make MIP nanoparticles an attractive tool for medical purposes, some adjustments are required in term of size, composition, and thus, synthesis technique. As described in Section 2.2, the precipitation polymerization technique led to particles with a size range limited to the range of precipitation which could be an issue for biomedical applications. For in vivo applications, particles with sizes above 200 nm are required to avoid a rapid elimination by the reticuloendothelial system and to favor EPR effect. Particles below 10 nm are rapidly removed through extravasations and renal clearance [59]. To overcome this particular issue while avoiding surfactant use, the hierarchical technique can be implemented: the target is bound to a porous silica gel (pore sizes about 11 nm) and the polymerization process takes place inside the pores. The size distribution is narrow compared to bulk materials (8–9 nm) and cross-links are homogeneously distributed [60]. However, the material presents less selectivity as the steric hindrance, caused by the binding to the silica surface, prevents complementary interactions to develop [61]. This technique has been adapted by Sellergren et al. in 2011 [62] for the imprinting of proteins (human serum albumin and immunoglobulin G) and can supposedly be used for applications in nanomedicine but to the best of our knowledge, none is currently under development.

The molecular imprinting process being cheaper than the use of antibodies to target a given molecule, it remains nonetheless expensive for biomedical applications as the cost of interesting proteins or drugs is often non-negligible. It is also well known that proteins can degrade in organic solvent and that their structure, a key factor in protein recognition and, hence, for an efficient protein imprinting, is altered by temperature or pH changes. The imprinting of proteins, beyond the difficulties regarding their stability, tends to produce higher cross-reactivity and insufficient extraction after the polymerization due to the size of the protein. Epitopes, small regions of a protein that are used as a recognition site, may be employed to address those particular issues [63,64,65].

**Figure 4:**
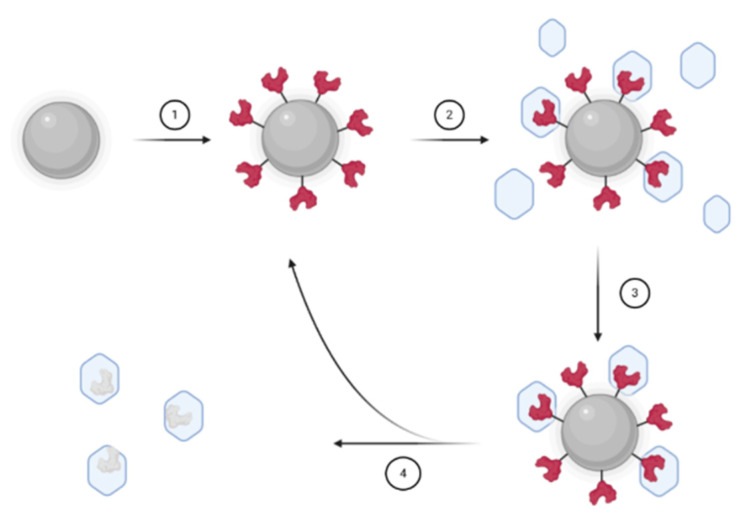
Solid phase synthesis technique: (1) the template is covalently coupled to glass beads, (2) a monomer mixture is added, polymerization is initiated, (3) non-specific polymers are washed using cool water, (4) release of specific polymers through hot washing, the swelling of the thermosensitive polymer allows the separation, the glass beads can be collected for a new polymerization. Created with BioRender.com the 3 April 2021.

Moreover, for medical applications, it is necessary to work with small and regular objects: specific methods have to be designed such as the solid-phase synthesis developed by Ambrosini et al. [66]. Instead of having the template in solution, the proteins are immobilized onto glass beads (100 µm) (step 1 Figure 4). The beads are then packed into a reactor with the reaction media containing monomers, initiator, and a crosslinker (step 2 Figure 4). After synthesis, the residual monomers and low affinity nanoMIPs are washed away (step 3 Figure 4) before extracting high-affinity nanoMIPs from the templates by hot washing or thermo-responsive swelling (step 4 Figure 4). Their attempt with trypsin resulted in high specificity and selectivity toward trypsin, without any surfactant used that could alter a protein conformation during the imprinting, and the release of the MIP from the functionalized glass beads is simply induced by a temperature change.

Immobilization of the template through an affinity ligand [67] or metal chelate functionalization of the glass-beads [68], instead of direct chemical attachment to the support, enables an oriented binding to the beads surface.

The solid-phase synthesis has been employed by other groups [69,70], with attempts to automatize the synthesis process and to improve the binding properties of the MIPs. This process increases the binding properties as non-imprinted nanoparticles are washed before releasing MIPs, less dialysis is required to remove the template and the reusable aspect of the glass beads [71] tends to decrease the cost of this technique. However, the glass beads suffer several drawbacks. First, their size being between 70 and 100 µm, the surface/volume ratio is not optimal, leading to a smaller number of proteins immobilized. Interactions between the template and the monomer were also limited by the fact that stirring has to be avoided as the beads are prone to abrasion, causing leaching of the template and undesired nanoglass particles.

**Figure 5:**
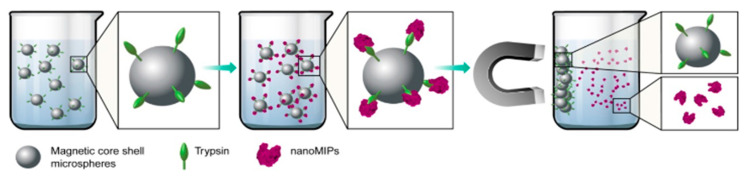
MIP solid-phase synthesis employing magnetic nanoparticles. Trypsin is immobilized on silica-coated magnetic nanoparticles prior to the synthesis. Reproduced with permission [72] © 2021 © The Royal Society of Chemistry.

As an alternative to address those issues, Sun et al. [72] replaced glass beads (70–100 µm) with magnetic microspheres (600–700 nm). Greater immobilization of trypsin was obtained, less solid material is employed and the nanoMIPs showed high affinity and selectivity for the template protein while the yield increased compared to conventional solid phase synthesis (Figure 5).

Solid-phase synthesis hence presents two main advantages compared to historical techniques that are really useful for medical applications: the fixation onto a surface, enabling more control over the orientation of the template during polymerization, and the glass beads ability to be reused, reducing the cost of the imprinting process. The possibility to automatize this process is also very interesting in the perspective of an industrialization of the production of this type of materials.

However, possible applications for those materials are limited by their inherent physical and chemical properties. Combining molecularly imprinting polymers to inorganic nanoparticles could lead to a novel class of hybrid materials with very interesting properties for nanomedicine, especially if they respond to external stimuli. As a support, those nanomaterials provide new synthesis pathways to those used for MIPs synthesis as described in the next part.

## 3. From MIPs to Hybrid MIPs

### 3.1. MIPs for Drug Delivery and Targeting

The very recent possibility to imprint larger molecules like proteins and to reduce the size of molecularly imprinted particles to nanoscale makes possible the in vivo application of those materials for medical purposes. For example, nanoMIPs can be used in many biological and medical fields, as drug delivery carriers or for other therapeutic purposes such as targeting.

**Figure 6:**
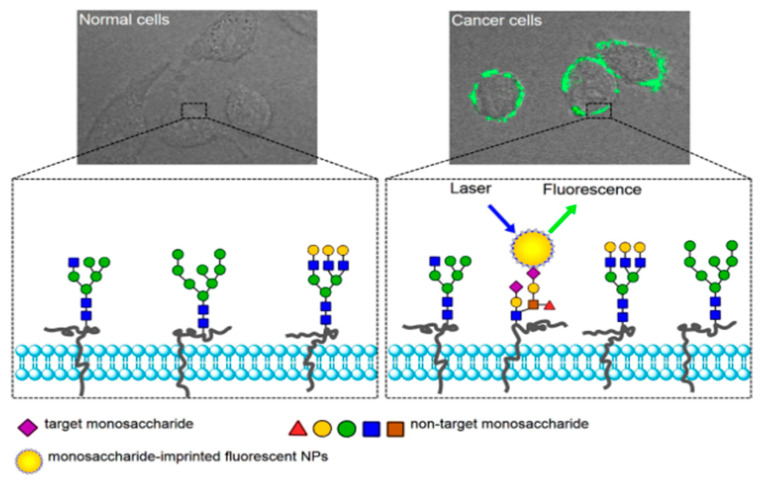
Targeting and imaging of cancer cells with monosaccharide-imprinted NPs. CC-BY license [73] Copyright 2016, Springer Nature.

As cancer cells tend to overexpress monosaccharides like mannose, fucose, or sialic acid on their surface, those make good targets for the imaging of cancer cells with MIP nanoparticles (Figure 6) [73].

In the case of a drug encapsulation inside a polymer matrix for passive delivery [74,75], the molecule is protected from enzyme degradation during its transit through the body thanks to the crosslinked polymer shell.

**Figure 7:**
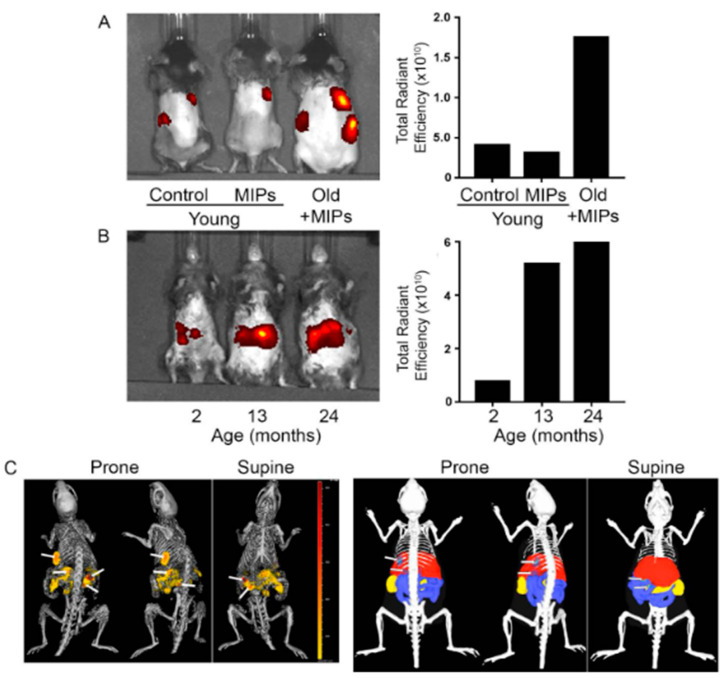
In vivo imaging of fluorescent B2M nanoMIPs. (**A**) Representative images of young (2 months) and old (15 months) mice injected intravenously with Alexa Fluor 647-tagged B2M MIPs. Control mice were injected only with vehicle. Animals were imaged in the prone position 2 h after injection. Total fluorescence signals were quantified and are shown in units of radiant efficiency. (**B**) Same as (**A**), with another group of mice of different ages, imaged in supine position. (**C**) 3D whole-body micro-CT (top) and fluorescence imaging tomography (FLIT, bottom) of the 15-month-old mouse in (**A**). Reproduced with permission [77] © 2021 The Royal Society of Chemistry.

It is also possible to combine passive drug release to targeting using MIPs as described by Piletsky et al. They developed an imprinted protein nanoparticle against Epidermal Growth Factor Receptor (EGFR), hence the material can specifically recognize a native protein and passively deliver a drug payload to the desired cell. The characteristics of molecularly imprinted polymers enables the access to in vivo targeting and imaging [76]. In 2019, Piletsky et al. developed fluorescent molecularly imprinted nanoparticles against an extracellular epitope of a biomarker, B2M [77], to detect senescent cells in vitro and in vivo. They showed that nanoMIPs do not elicit toxic responses in the cells or in mice and successfully recognize old animals, which have a higher proportion of senescent cells in their organs (Figure 7). Importantly, they loaded nanoMIPs with drugs and showed that they can specifically kill senescent cells via passive diffusion.

However, in order to keep the drug inside the polymer and actively deliver the payload only at the desired time and place, it is possible to combine MIPs with inorganic cores displaying interesting physical properties.

### 3.2. Properties of Inorganic Particles

Since the beginning of the 2000s, inorganic nanoparticles have been actively investigated as carriers for cell delivery [78]. Inorganic nanoparticles combine several advantages: a surface/volume ratio that enables the grafting/adsorption of large quantities of drugs and at the same time a good colloidal stability necessary for intravenous injection. However, what makes them very attractive for nanomedicine applications is their physical properties which depend on the composition of the nanoparticle. Among the most studied, one can find gold nanoparticles for their plasmonic properties, iron oxide which are superparamagnetic, silica particles which can be functionalized in a controlled manner, and quantum dots exhibiting luminescent properties.

Gold nanoparticles have been widely explored as therapeutic agents for displaying several coveted properties. In cancer therapy, it is possible to use gold nanoparticles as antineoplastic agents as they are capable of altering cell cycles [79] through their capacity to enhance radiation sensitivity and vascularization processes [80] thanks to their intrinsic property: they can interact selectively with heparin-binding glycoproteins and inhibit their activity. Gold nanoparticles may also be used for photothermal therapy: the excitation of surface plasmon oscillation through light exposition generates local heat release around nanoparticles [81]. This property can be used for cancer therapy [82,83] as evaluated in clinical trials. For instance, Nanospectra Biosciences trial, Aurolase, treated lung cancer with gold nanoparticles and laser radiation delivered through bronchoscopy. The coupling of gold nanoparticles with molecularly imprinted polymers may lead to new triggered release techniques using thermosensitive polymers and the photothermal properties of the particles.

Another class of nanoparticles that would provide interesting properties for MIPs hybrids is superparamagnetic iron oxide nanoparticles (SPIONs). Due to their size and superparamagnetic properties, strong magnetization at low magnetic field without remanence when the field is off, SPIONs may be employed as MRI contrast agents. For liver imaging, two solutions, approved by the FDA after 1996, were commercially available: Feridex^®^ and Resovist^®^, now no longer employed [84]. Superparamagnetic particles are currently more studied for their ability to magnetically guide carriers in vivo or generate heat upon alternating magnetic field (AMF) stimulation, so-called magnetic hyperthermia [22]. NanoTherm^®^ (MagForce Ag) is a SPION based technology using magnetic hyperthermia properties [84], approved in Europe for the treatment of glioblastoma and currently evaluated in the USA for the treatment of prostate cancer.

It is hence possible to combine such nanoparticles with thermosensitive polymers for a triggered drug release or cell-targeted hyperthermia therapy. The most widely employed magnetic material to be coupled to MIP is magnetite, Fe_3_O_4_, due to its excellent characteristics, such as low toxicity, easy synthesis, and low production cost. MIP may also be coupled to maghemite, γ-Fe_2_O_3_, less employed due to the additional oxidation step required but presenting the same main characteristics as Fe_3_O_4_ on top of being chemically more stable. The main challenges with those particles are long-term chemical stability and functionalization. To address those issues, it is possible to add an organic (polymer, dendrimer) or inorganic (silica, gold) shell before the MIP functionalization which will provide a protection against oxidation as well as new functionalization pathways.

Another interesting property that can be added to MIP is fluorescence emitted by quantum dots. A quantum dot (QD) is a nanosized crystal of inorganic semiconductor that has size-tunable, narrow, Gaussian emission spectra excited at a single wavelength, enormous absorption extinction coefficients, and high fluorescent quantum yields. They are inorganic, photochemically robust, resistant to photobleaching, and exceptionally bright. Finally, quantum dots “blink”, which ensures the observation of a single dot event, translating to the observation of a single protein. It is possible to design biocompatible QD for in vivo applications by modifying the surface of the usual CdSe, CdTe, or ZnSe nanocrystals or through conjugation with antibodies, peptides, or small molecules [85,86]. They have been widely developed for bioimaging and drug delivery [87], hence they make good candidates for the synthesis of hybrid MIPs for medical application as a great number of publications already support such combination in the design of sensors [88,89,90]. Carbon dots (CDs) may provide similar results. Carbon dots are small nanosized particles (3 nm) that exhibit remarkable photoluminescent properties. They are chemically inert, without heavy metals and more photostable than dyes and other quantum dots.

**Figure 8:**
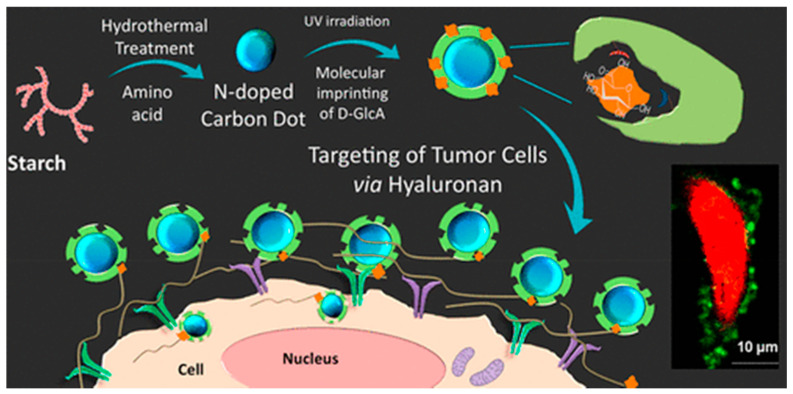
Synthesis and mode of action of CD coupled to a MIP coating to target tumor cells. Reproduced with permission [93] © 2021 American Chemical Society.

To synthesize those hybrid materials, it is possible to do a bulk-like polymerization, where the template is added with the monomer and the core, or to combine covalent and non-covalent approach by grafting the template on an inorganic surface such as silica nanoparticles [91,92]. The latter, as described by Liu et al. [73], requires additional steps to ensure the template immobilization prior to polymerization. This method is less time-efficient but is preferred if a specific orientation of the template is necessary or if the one-pot process is not successful. In the case of sialic acid imprinted polymer described by Liu et al. [92], no MIP layer is formed using the bulk process, probably due to a lower pH induced by the sialic acid, slowing down the polymerization process, but pre-immobilization of the template solved this particular issue. Additionally, the intrinsic properties of the nanoparticles may be exploited during the synthesis: the luminescence of the CDs can be used as a light source for local photopolymerization, resulting in thin layers of MIP coating the fluorescent CDs (Figure 8) [93].

**Figure 9:**
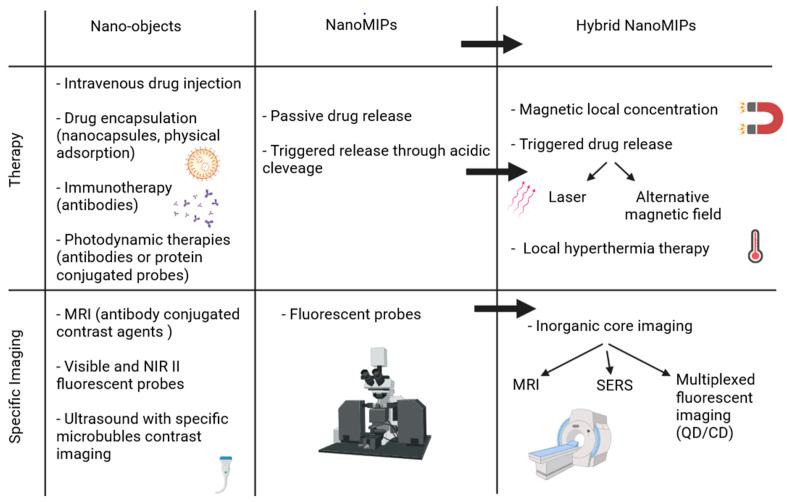
Non-exhaustive comparison of possible applications for therapy or bioimaging between usual nano-objects, molecularly imprinted polymers, and their hybrid counterpart.

A wide range of new applications for molecularly imprinted polymers can be developed when they are combined to inorganic materials but the grafting of MIPs onto the surface of inorganic nanoparticles also results in core-shell structures that allow more control over the size and distribution of the material. The main improvements provided by nanoMIPs and hydrid nanoMIPs compared to other nano-objects is non-exhaustively described in Figure 9.

### 3.3. Toxicity and Stealthiness

The combination of a polymer matrix with nanostructures usually provides additional properties [94]. However, the complexity of a material may increase the number of possible side effects. The incorporation of inorganic nanoparticles that are themselves already suspected to induce some toxicity [95,96] has to be considered when evaluating the benefice/risk balance of such innovation.

For in vivo applications, the biocompatibility of the hybrid material as well as its degradation characteristics are to be studied. For instance, Griffete et al. [97] studied the biological fate of iron oxide molecularly imprinted polymer-coated nanoparticles. In particular, they evaluated the impact of the acrylamide polymer coating on the degradation of iron nanoparticles in a lysosome-like buffer and in a model of cartilage tissue formed by differentiated human mesenchymal stem cells. They found that the polymer coating tends to slow down the iron nanoparticles degradation without hindering it or affecting the internalization of the nanoparticles. This aspect is encouraging for the development of nanomedicine tools involving hybrid materials.

For therapeutic purposes, the endocytosis by macrophages is also a key factor, as an excess of MIPs endocytosis decreases the amount of circulating particles, increasing the amount needed for a therapeutic effect. Dong et al. [24] evaluated the cytotoxicity and the macrophage endocytose of SiO_2_-MIPs hybrid nanoparticle targeted against HER2, a receptor overexpressed in some types of breast cancer cells. Their material, while efficiently targeting breast cancer cell HER2+ and inhibiting tumor growth, proved to be non-cytotoxic and showed only a small macrophage uptake and is hence very promising as a new strategy against breast cancer.

However, it has to be noted that cells experiments, as a model, are limited and that more accurate results, obtained through small animal experiments, will be required to assess the potential risk of those materials.

## 4. Application of Hybrids nanoMIPs for Medicine

### 4.1. Bioimaging

As described in Section 2.1, the recognition properties of MIPs are useful in the medical field to target specific tissues, cells, or compounds. It is therefore essential to be able to image the targeted areas. The imaging performances can be greatly improved combining the MIP technology with inorganic materials. With this goal, fluorescent proteins or antibodies can be replaced by their fluorescent MIP counterpart for those applications. For instance, epifluorescence and confocal microscopy can be used for live cell imaging [98]. NanoMIPs can promote the development of cell imaging tools against difficult targets such as membrane proteins as well as intracellular proteins and can even target complex structures such as whole cells [99]. The use of quantum dots for instance renders accessible new ranges of emission and excitation wavelength, farther from the autofluorescence of biomolecules, improving the signal/noise ratio. In 2017, Cecchini et al. [100] first targeted the human vascular endothelial growth factor (hVEGF), overexpressed in many invasive cancers, using molecularly imprinted polymer nanoparticles. They developed nanoMIPs with quantum dots embedded, able to selectively bind to hVEGF while displaying a characteristic signal for bioimaging. Their ability to specifically target hVEGF was confirmed in xenotransplantation of human malignant melanoma cells expressing hVEGF in zebrafish embryos.

**Figure 10:**
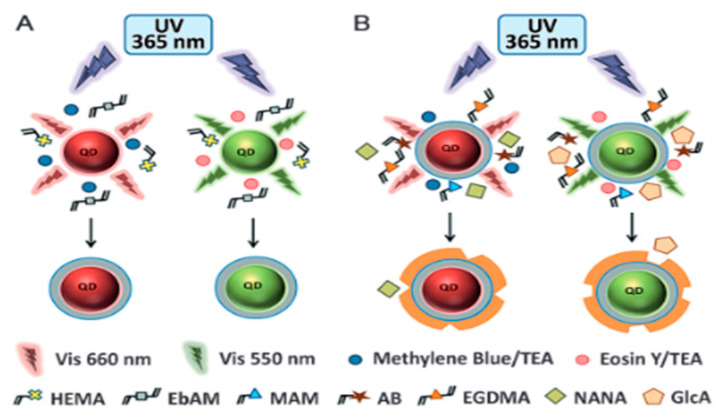
(**A**) Red or green light emitted from InP/ZnS quantum dots excited by UV irradiation is used to synthesize a polymeric shell in situ around the particles by photopolymerization. Methylene blue/triethyl-amine (TEA) are used as the initiator system for red-QDs and eosin Y/TEA for green-QDs. (**B**) A second shell of MIP is synthesized by reinitiation in the presence of functional and cross-linking monomers and a molecular template (GlcA or NANA). Reproduced with permission from [87] Copyright 2016 John Wiley & Sons.

The combination of MIPs with QDs was described for multiplexed cell imaging exploiting the size-tunable emission of QDs through the combination of various templates with distinct QDs. A multiplexed imaging technique combining MIP-coated InP/ZnS quantum dots and non-hybrid MIP containing a fluorescent rhodamine monomer was developed in 2017 by Haupt et al. [101]. First, a hydrophilic coating was synthesized onto the QD nanoparticles to ensure water-compatibility for the MIP layer synthesis. Then, a MIP layer was formed with glucuronic acid (GlcA) or N-acetylneuraminic acid (NANA) as a template, making the material able to efficiently target the extracellular and intracellular hyaluronan and sialylation sites (Figure 10).

In 2017, hyaluronan and salicylic acid nanoMIPs were employed as potential indicators of pathological condition to localize hyaluronan and sialylation sites on fixed and living human keratinocytes [102]. The quantum dots coated with MIPs were non-cytotoxic and did not affect cell viability or morphology, offering a promising tool for bioimaging on living tissue.

**Figure 11:**
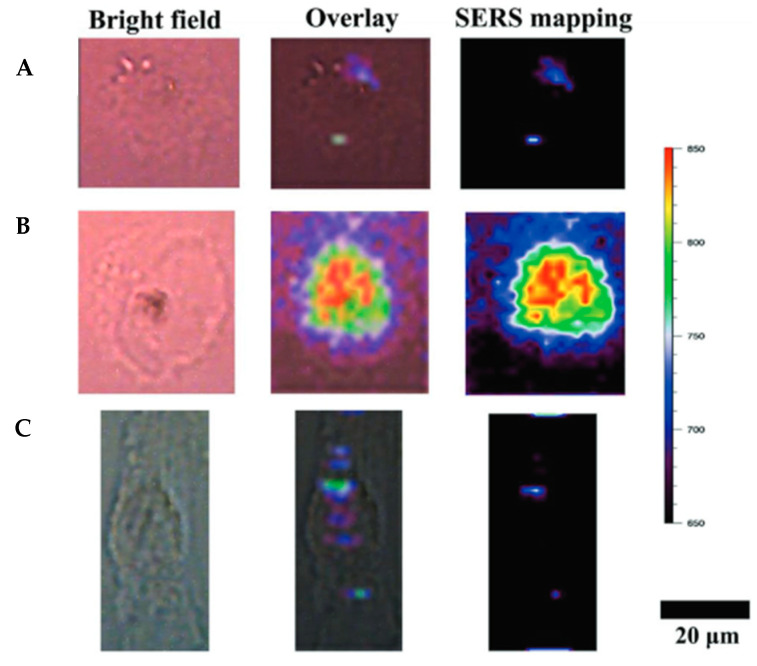
Confocal Raman images of MCF-7 breast cancer cells stained with (**A**) NIP@AuNR and (**B**) MIP@AuNR against EGFR. (**C**) Wpmy-1 human normal prostate stromal cells stained with MIP@AuNR. Reproduced with permission from [94] © 2021 John Wiley and Sons.

Hybrid MIPs may also be employed as surface-enhanced Raman scattering (SERS) tags for bioimaging [94]. First developed by Yin et al. [103] to target cancer cells, their method consists in the imprint of sialic acid onto Raman-active silver nanoparticles. After laser excitation, the resulting SERS signals from the MIP nanoparticle were able to differentiate cancer cells and tissues from normal ones. Similarly, gold nanorods coupled to EGFR-imprinted polymers have been employed for live cell Raman imaging [94] and could be employed for bioimaging (Figure 11).

### 4.2. Therapy

As MIPs can selectively and specifically adsorb molecules, they can be used for passive or active drug delivery.

**Figure 12:**
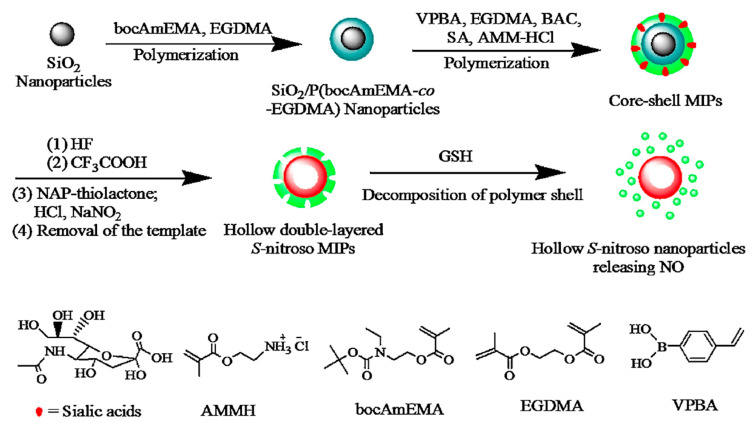
Synthesis method of sialic acid imprinted hybrid nanoparticles. The intracellular degradation of the sulfur bonds of the polymer in presence of GSH at low pH generate nitrous oxide with anticancer properties. Reproduced with permission from [92] © 2021 Elsevier B.V.

The most impressive advances toward controlled release have been obtained through the combination of MIPs with inorganic materials. Sometimes, the inorganic part only serves as a support for a clever release mechanism such as developed in 2016 by Zhang et al. [104]: a silica-coated MIP encapsulating DOX and containing sulfur–sulfur bonding. In acidic pH and high concentration of glutathione (GSH), the S-S bonds break, facilitating drug release. Similar mechanisms were then exploited in 2018 by Yang et al. [92] in a material where the silica core is removed by degradation of the S-nitrosothiol polymer by GSH at low pH generated nitrous oxide which acted as an anticancer drug (Figure 12).

Beyond facilitating the synthesis, the addition of inorganic particles often provides new tools for triggered drug release.

In 2014, UV was used to trigger drug release from asymmetric MIP-silver nanoparticles (Janus particles) synthesized via a wax−water Pickering emulsion. The emulsion allowed a one-sided silver coating of MIPs nanoparticle. Release of the drug from the Janus MIP particles is controlled by switching on−off the UV illumination [105]. However, this system lacks biocompatibility due to the application of Ag NPs and the limitation of UV illumination which damages cells. Therefore, adjustments are required for practical applications. For example, we could imagine replacing Ag NPs by gold nanorods which are nontoxic and more biocompatible.

Magnetic MIP have also emerged as a powerful material for controlled drug delivery systems because they can be localized to the delivery sites using a magnet [106] and release the drug to particular sites through passive diffusion or polymer degradation. For instance, magnetic molecularly imprinted polymers were synthesized for special recognition and slow release of aspirin [107]. The synthesized MIPs have a high magnetic responding capacity, which enables them to be separated from suspension by an external magnetic field. The resulting magnetic MIPs exhibit good special binding and selectivity capacities to the template molecule.

**Figure 13:**
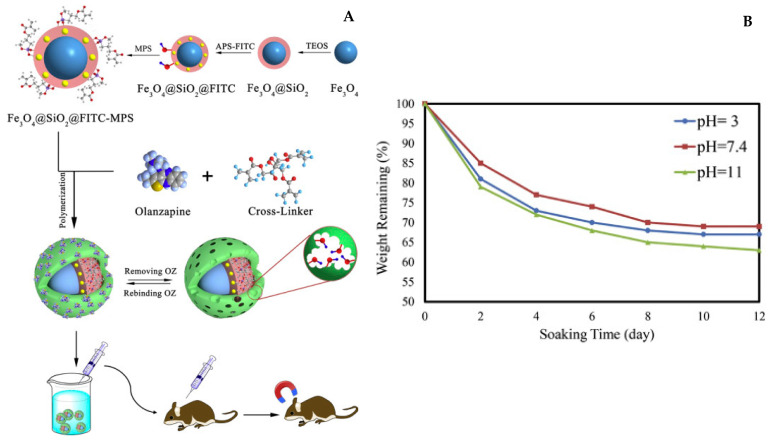
(**A**) An iron oxide core is coated with silica and functionalized with a fructose polymer imprinted with olanzapine, an anti-psychotic drug. (**B)** Drug release occurs naturally in the body as the polymer is degraded in a wide range of pHs after a few days. Reproduced with permission from [106] © 2021 Elsevier B.V.

Drug release can also be induced by polymer degradation as Asadi et al. [106] illustrated with their Fe_3_O_4_-modified MIP nanoparticles containing olanzapine (Figure 13). In particular, they showed that the drug release can occur at pHs close to live body environment as the fructose-based polymer is degraded.

However, the main interest of the molecularly imprinted polymers combination with maghemite/magnetite resides in their core processing photothermia [108] and hyperthermia properties [22]. While most studies focus on passive delivery, it is possible to use the properties of magnetic nanoparticles to trigger the delivery of a drug or to kill specific cells through heat exposure.

**Figure 14:**
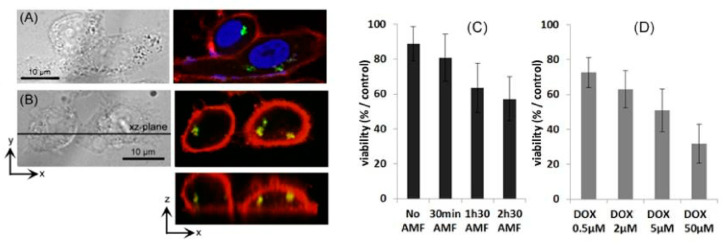
Cancer cells (PC-3) internalization of the Fe_2_O_3_@DOX-MIP nanoparticles. DOX is detected in the green channel, nuclei and cell membranes are stained by DAPI (**A**) in blue and PKH26 (**A**,**B**) in red. Z reconstructions (**B**) identify DOX inside the cells. (**C**): Viability of cancer cells labeled with Fe_2_O_3_@DOX-MIP after exposure to the alternating magnetic compared to the control experiment. (**D**) Treatment of the cancer cells with free doxorubicin (DOX) incubated for 2 h from 0.5 to 50 μM. Reproduced with permission from [109] © 2021 RSC Publishing.

In this context, in 2015, an innovative magnetic MIP nanomaterial for triggered cancer therapy showing active control over drug release by using an alternating magnetic field was developed [109,110]. Upon AMF exposure, the hydrogen bonds between the MIP and the encapsulated drug, doxorubicin, are broken and the molecule is released without any significant heating of the medium. After AMF application, cancer cell viability is reduced to 60% after 1 h 30 min treatment in athermal conditions while the control cells do not suffer any mortality (Figure 14). The same behavior was obtained with magnetic silica imprinted polymer [111].

Other groups have worked on triggered systems that could release a drug upon a signal while avoiding burst effects. In this purpose, Yan et al. developed a molecularly imprinted polymer doped with graphene oxide quantum dots [112] enabling the controlled release of DOX through the photothermal properties of QDs. The prepared polymer micro-spheres doped with graphene QDs via miniemulsion polymerization using a polymerizable ionic emulsifier were efficiently photothermally triggered to release DOX while avoiding any leakage of the drug.

### 4.3. Theranostic

New systems combining both aspects of MIPs—the ability to efficiently target and to encapsulate therapeutic molecules to be delivered—are starting to spread in the theranostic field. The main idea is to combine a biological marker characteristic of a pathological condition as a target and a therapeutic molecule to enhance the potency of a treatment while imaging and reducing potential side effects. This approach has been widely used in cancer therapy as drugs employed in chemotherapy are also toxic for healthy cells, resulting in acute side effects.

The recent development of technologies combining molecularly imprinted polymers with inorganic core materials is so versatile that it allows a wide range treatment option. In particular, those materials could specifically target any given tissue or cell types that possess a specific marker and that such marker possesses the required stability to be imprinted. Like non-hybrid materials, they can be combined with a drug loading inside the polymer matrix but additional features are available when using an appropriate core material. The possibilities seem endless and are starting to be broadly explored in the literature.

In 2019, Zhang et al. [113], developed an innovative silica-based material that could be used for targeting, drug delivery, and imaging. A biomarker, human fibroblast growth-factor-inducible based, coupled with an anticancer, bleomycin, were imprinted on a silica core for its good optical properties. In vivo experiments showed an excellent inhibition of the growth of BxPC-3 xenograft tumor. Similar models have been developed against other cancer cells, for instance one targeting the HER2 receptor and delivering DOX [114]. It led to similar results in vivo, illustrating the versatility of this approach.

It is also possible to use the magnetic properties of a material to selectively target an organ or a tumor instead of imprinting a surface marker. The ability of SPIONs to accumulate in a specific site through magnetic guidance has been successfully demonstrated in a study without the imprinting of molecules [115]. In a recent study, a 5-fluorouracil, anticancer drug with fast degradation rates, loading novel multi core–shell structure nanocarriers based on a cross linker of tannic acid has been synthesized [116].

**Figure 15:**
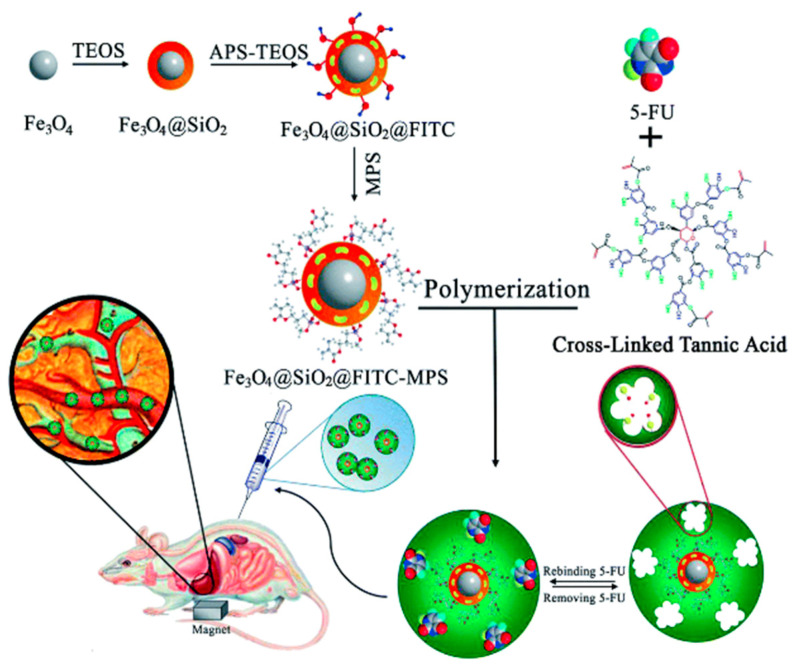
The procedure followed for preparation and characterization of magnetic molecularly imprinted polymer (MMIP) nanoparticles as a 5-FU carrier (TEOS: tetraethyl orthosilicate, APS: 3-aminopropyl trimethoxysilane, FITC: fluorescein isothiocyanate, 5-FU: 5-fluorouracil, MPS: methacryloxypropyl trimethoxysilane). Reproduced with permission from [116] © 2021 The Royal Society of Chemistry.

Fluorescent imaging with a small animal imaging instrument confirmed the successful conduction of the carrier into the liver by applying an external magnetic field. However, as liver and kidney tend to naturally accumulate nanoparticles, the choice of liver located tumors might not be the most appropriate to illustrate an efficient targeting (Figure 15).

## 5. Conclusions

In the past, molecularly imprinted polymers have been mostly used in sensors or for analytical purposes but during the last decade, great developments have been made in order to use them in nanomedicine. The synthesis techniques have evolved in order to ensure the biocompatibility of the material, to control the size or even the orientation of the imprint. As MIP nanoparticles are cheaper than antibodies, with an increased stability and more adapted to mass production with alternatives to reuse the template proteins [117], they represent a particularly good alternative for targeting applications for bioimaging or for therapy. However, if the particles are used in vivo, it is necessary to keep in mind that a protein corona can coat MIPs, decreasing their capacity to recognize the targeted protein by preventing access to the imprint inside the polymer as studied by Lagana et al. in 2020 [118]. Hence, special attention needs to be placed on the future development of MIPs for nanomedicine. If protein imprints are employed for in vivo targeting, some solutions have to be developed and implemented in order to control protein corona formation.

Additionally, in order to obtain novel interesting properties, they can be combined with inorganic materials such as iron oxide, gold, or silver nanoparticles, which have the capacity to respond to external physical stimuli. Fluorescence imaging has also become a fundamental tool for biomedical applications. In the past decade, the fluorescence in the second near-infrared window (NIR-II, 1000–1700 nm) has been developed to achieve deep penetration and thus significant biomedical applications have begun to emerge [119]. Hence, novel synthetic strategies are to be directed toward the development of hybrid imprinted nanoparticles for nanomedicine to exploit those advances. In particular, the imprinting of proteins, often required for medical applications, is usually more complex than imprinting small stable molecules. However, even if there are several hybrid MIPs that exist, as listed in Table 1, they are scarcely used for their physical properties. For example, combining a MIP that can recognize the imprint of a cancer marker, to inorganic cores that heat in response to an external stimulus will produce an effective material for selective cancer destruction. Nanoparticles such as gold have been widely used in cancer phototherapy and photoimaging, owing to their enhanced solubility, stability, biofunctionality, cancer targetability, and biocompatibility [120]. All-in-one materials, combining targeting, imaging, and drug delivery, seems to be within grasp through the combination of MIPs with inorganic nanoparticles. Many interesting combinations will be released in the next few years that will provide an extensive panel for patient care.

## Figures and Tables

**Table 1:** Hybrid molecularly imprinted nanoparticles applied to nanomedicine.

Inorganic Material	Monomers	Synthesis Technique	Target	Application	Ref
CdTe QDs	N-isopropylacrylamide, N-tertbutylacrylamide, N(3-aminopropyl) methacrylamide hydrochloride acrylic acid N,N’methylenebisacrylamide	Solid phase synthesis of MIPs and chemical coupling to QDs	hVEGF	Bioimaging	[100]
Fe_2_O_3_	acrylamide, ethylene glycol dimethacrylate	Bulk thermopolymerisation	Doxorubicin	dds	[109]
Fe_2_O_3_	Acrylamide, N,N-methylene-bis-acrylamide	Bulk redox polymerization	GFP	Targeting/drug delivery	[97]
Fe_3_O_4_	methacrylic acid, trimethylolpropane trimethacrylate	Bulk polymerization	Aspirin	dds	[107]
Fe_3_O_4_/SiO_2_	Fructose	Co-precipitation polymerization	Olanzapine	dds	[106]
Fe_3_O_4_/SiO_2_	Tannic acid	Mini-emulsion polymerization	5 fluorouracil	dds	[116]
FITC doped SiO_2_ NPs	TEOS	Bulk polymerization with pre-oriented template	HER2	cancer therapy	[24]
FITC-doped SiO_2_ NPs	TEOS	Template immobilization (boronic acid)	Sialic acid, fucose or mannose	Fluorescence imaging (FITC)	[73]
gold nano rods		Poly(NIPAAm) template adsorption at 15 °C and colapsing at 37 °C generating an imprint	EGFR	Bioimaging	[94]
Graphene oxide QD	Methylmetacrylate, ethylene glycol dimethacrylate	Mini-emulsion polymerization	Doxorubicin	dds	[112]
InP/ZnS QD	4-acrylamidophenyl)(amino) methaniminium acetate, methacrylamide, ethylene glycol dimethacrylate or 2-hydroxyethyl methacrylate,N,N’-ethylenebis(acrylamide)	Bulk photopolymerization using QD’s emission	Glucuronic acid or N-acetylneuraminic acid	Bioimaging	[87]
silica	EGDMA	Bulk polymerization	Doxorubicin	dds	[104]
silica	Dopamine	Bulk dopamine condensation	HER2 + doxorubicin	dds/targeting	[114]
silicon nanoparticles	Ethylene glycol dimethacrylate, zinc acrylate, 4-Vinylbenzeneboronic acid	Bulk polymerization	Bleomycin + human fibroblast growth-factor-inducible 14	dds/targeting	[113]
silver	TEOS	Boronate affinity-oriented surface imprinting approach/TEOS condensation	Sialic acid	Bioimaging (RAMAN)	[103]
silver	Methacrylic acid, N-isopropylacrylamide, N,N’-methylene-bis-acrylamide, allylamine	Wax in water Pickering emulsion for the Ag coating	propanolol	UV dds	[105]
Starch-based CD	4-acrylamidophenyl)(amino)methaniminium acetate, methacrylamide, ethylene glycol dimethacrylate	Bulk photopolymerization	Glucuronic acid	Cancer cell targeting and imaging	[93]

NIPAm: N-isopropylacrylamide, BIS: N,N’-ethylenebis(acrylamide), AMPA: N-(3-aminopropyl) methacrylamide hydrochloride, EGDMA: ethylene glycol dimethacrylate, TEOS: Orthosilicate de tétraéthyle, MA: Methacrylic acid, MMA: Methylmetacrylate, AB: 4-acrylamidophenyl)(amino)methaniminium acetate.

## Abbreviations

AFM	Atomic force microscope
AMF	Alternative magnetic field
ASP	Aspirin
CD	Carbon dot
DOX	Doxorubicin
EGDMA	Ethylene glycol dimethacrylate
EGFR	Epidermal growth factor
ELISA	Enzyme-linked immunosorbent assay
EPR effect	Endothelial permeability and retention effect
FTIR	Fourier transform infrared spectroscopy
hVEGF	Human vascular endothelial growth factor
LOD	Limit of detection
MAA	Methacrylic acid
MIP	Molecularly imprinted polymer
MRI	Magnetic resonance imaging
NIP	Non-imprinted polymer
NIR	Near infrared
NP	Nanoparticle
QCM	Quartz crystal microbalance
QD	Quantum dot
SAW	Surface acoustic wave
SERS	Surface enhanced Raman spectroscopy
SPIONs	Superparamagnetic iron oxide nanoparticles
SPR	Surface plasmon resonance
TEM	Transmission electronic microscopy
